# Measurement-Device-Independent Two-Party Cryptography with Error Estimation

**DOI:** 10.3390/s20216351

**Published:** 2020-11-07

**Authors:** Zishuai Zhou, Qisheng Guang, Chaohui Gao, Dong Jiang, Lijun Chen

**Affiliations:** 1 State Key Laboratory for Novel Software Technology, Nanjing University, Nanjing 210046, China; mf1833107@smail.nju.edu.cn (Z.Z.); mg1733016@smail.nju.edu.cn (Q.G.); dz1833008@smail.nju.edu.cn (C.G.); jiangd@nju.edu.cn (D.J.); 2 School of Internet, Anhui University, Hefei 230039, China

**Keywords:** quantum, two-party cryptography, measurement-device-independent

## Abstract

We present an innovative method for quantum two-party cryptography. Our protocol introduces joint measurement and error estimation to improve the security of two-party cryptographic protocols. Our protocol removes the assumption of the attacker’s limited power and catches the attacking actions through highly estimated bit error rate. Our protocol is formally proved to be secure against both eavesdroppers and dishonest communication parties. We also utilize our designed protocol to construct two specific two-party cryptographic applications: Quantum bit commitment and quantum password identification.

## 1. Introduction

Two-party cryptographic protocol is a significant branch of modern cryptography. It can realize communication between mutually distrustful parties [1,2,3]. However, the advent of a quantum computer will pose a huge threat to cryptographic protocols that originally rely on computational complexity. Fortunately, Bennett and Brassard proposed the first quantum cryptographic protocol in 1984, known as BB84 quantum key distribution (QKD) protocol [1]. BB84 protocol allows two mutually trusted parties to generate identical secret keys for encryption. Quantum cryptography, laying its foundation on quantum mechanics, can provide unconditional security in the communication process. Therefore, studies over quantum cryptography have aroused worldwide attention.

While QKD has gained extensive concern nowadays, researchers also consider introducing quantum technology into two-party cryptographic protocols. However, Lo and Mayers independently demonstrated that unconditionally secure two-party cryptographic protocol does not exist without restricting the attacker’s ability [4,5,6,7]. Therefore, a perfect two-party cryptographic protocol is more difficult to be realized than key distribution. Even so, several solutions were proposed to seek more secure quantum two-party cryptographic schemes, among which there are mainly three types. The first solution introduces the relativity theory to restrain attacker’s behavior [3,8,9,10]. The second solution weakens the demand for security. In other words, it gives up the pursuit of perfect security and allows the attacker’s behavior to succeed with negligible probability. The most representative example is the cheat-sensitive quantum bit commitment (CSQBC) protocol [11,12,13,14]. The third solution is limiting the attacker’s power to current technologies. For example, in 2005, Damgård demonstrated secure two-party cryptography under the assumption that the attacker’s capability of storing quantum states was limited. In this so-called bounded storage model [15,16], the attacker is equipped with perfect quantum storage, but the storage capacity is limited because of unaffordable cost. Later, Schaffner extended the model to the noisy storage model [2,17], where the attacker possesses quantum storage with unlimited capacity, but the noise increases over time.

Although Konig manifested secure two-party cryptographic protocols are feasible under noisy bounded model [2,17], we are still interested in designing two-party cryptographic protocols when the attacker possesses perfect quantum storage inspired by He’s work [18]. We have discovered that two-party cryptographic protocols, like bit commitment, oblivious transfer, in Ref. [2,11,17] do not have the process of error estimation, which serves as a significant indicator of eavesdropping attack in QKD. The reasons are obvious: For one thing, the communication parties do not trust each other and for another, the information is asymmetric between parties during the communication process.

Inspired by the foundations of measurement-device-independent QKD (MDI-QKD) [19,20,21] and phase-matching QKD (PM-QKD) [22,23], we make it possible to introduce error estimation into two-party cryptographic protocols. In the QKD process, once there is an eavesdropper, the final key error rate will exceed the upper limit. Therefore, in encrypted communication between the two parties, if one party is dishonest, the information they previously negotiated will also have a higher error rate, which is difficult in avoiding detection by another party. In MDI-QKD, the measurement stage is independent of the final key. This de-emphasizes the assumptions for the attacker’s quantum memory and enables us to discover the attacker by the increased quantum bit error rate during the estimation process.

In this paper, we introduce joint measurement method in MDI-QKD and PM-QKD, and error estimation into two-party cryptographic protocols, and raise our improved weak string erasure (WSE) protocol and 1-2 random oblivious transfer (ROT) protocol. These two protocols are significant for other TPC (two-party cryptographic protocols) applications. Compared with existing WSE and 1-2 ROT protocol, our protocol does not make any assumption on the attacker’s devices. Instead, we restrict the attacking behavior by the protocol itself, which offers greater security. In our protocol, the honest party does not need quantum storage devices and the devices are compatible with mainstream QKD platforms.

The paper is organized as follows. Section 2 introduces the foundations of our research. Section 3, and Section 4 discuss our proposed WSE and 1-2 ROT respectively, and demonstrate their security. In Section 5, we probe into applications of two-party cryptographic protocols and propose two important practices, quantum bit commitment and password-based identification. Finally, the paper ends with a conclusion.

## 2. Preliminaries

This section will introduce several fundamental concepts to our research, including entropy qualities, joint measurement, error estimation, and privacy amplification.

This paper follows the notations in Ref. [2], using [n]:={1,2,…,n} for the set of nature number, and $2^{\left[n\right]}:=\left\{S|S\subseteq \left[n\right]\right\}$ is the set of all possible subsets of [n].

### 2.1. Entropy Qualities

Here, we present some crucial entropy qualities for our security proof. Bulleted lists look like this:

**Definition** **1** (Shannon entropy)**.** *P(X) is the probability distribution function of a random variable X. The entropy H(X) is defined as:*

$$H\left(X\right)=-\sum_{X}P\left(x\right)\log_{2}P\left(x\right).$$

As same as Ref. [2,19], we define guessing probability:

$$p_{\mathrm{guess}}\left(X|E\right)=\max_{M_{x}}\sum_{x}P_{X}\left(x\right)\mathrm{Tr}(M_{x}\rho_{E}^{x}),$$

where $p_{\mathrm{guess}}\left(X|E\right)$ is the probability of guessing *X* when given register *E*, and its maximization is over all positive operate-valued measurements (POVMs) {$M_{x}$} acting on register *E*. Then we can easily get that the conditional min-entropy of *X* given *E* is:

$$H_{\min}\left(X|E\right)=-\log_{2}p_{\mathrm{guess}}\left(X|E\right),$$

and also the definition of conditional smooth min-entropy is:

$${H_{\min}}^{\varepsilon }\left(X|Y\right)=\max_{E}H_{\min}\left(XE|Y\right),$$

where for any event E, we have:

$$p_{\mathrm{guess}}\left(XE|Y\right)=\sum_{y}P_{Y}\left(y\right)\max_{x}P_{XE|Y}\left(x|y\right).$$

Next, we discuss min-entropy-splitting lemma used in Ref. [2,17] for the security proof of 1-2 ROT and WSE protocol.

**Lemma** **1** (Entropy splitting [2,17]). *Let ε≥0, and $X_{1},X_{2},\cdots ,X_{m}$ and Z are random variables subjected to $H_{\min}^{\varepsilon }\left(X_{i}X_{j}|Z\right)\geq \alpha$ (i≠j). There exists a random variable V⊆{1,⋯,m} such that for any independent random variable W⊆{1,⋯,m} with $H_{\min}\left(W\right)\geq 1$,*

$$H_{\min}^{2m\varepsilon }\left(X_{W}|VWZ,V\neq W\right)\geq \frac{\alpha }{2}-\log_{2}\left(m\right)-1.$$

**Lemma** **2** (Min-entropy splitting [2,17]). *Let ε≥0, and $X_{0}$, $X_{1}$, and Z are random variables subjected to $H_{\min}^{\varepsilon }\geq \alpha$. Then there exists a random variable D∈{0,1}, such that:*

$$H_{\min}^{\varepsilon }\left(X_{D}|DZ\right)\geq \frac{\alpha }{2}-1.$$

Finally, we introduce quantum uncertainty relation as the core of security proof for our redesigned protocol.

**Theorem** **1** (Quantum uncertainty relation [24]). *Suppose Q is an arbitrary fixed n-qubit state, and θ is a random basis ($\theta \in_{R}\left\{0,1\right\}$), and $X\in_{R}{\left\{0,1\right\}}^{n}$ is a random variable for the outcome of measuring Q in basis $\theta^{n}$, then it has δ>0, and the conditional smooth min-entropy has a lower bound such that:*

$$H_{\min}^{\varepsilon }\left(X|\theta^{n}\right)\geq \left(\frac{1}{2}-2\delta \right)n.$$

*Here,*

$$\varepsilon =2\exp\left(-\frac{{\left(\frac{\delta }{4}\right)}^{2}}{32{\left(2+{log}_{2}\frac{4}{\delta }\right)}^{2}}\right).$$

### 2.2. Joint Measurement

Joint measurement and phase-matching are widely used in QKD, and we introduce them to our two-party cryptographic protocol. Next, we explain these two methods.

**Figure 1:**
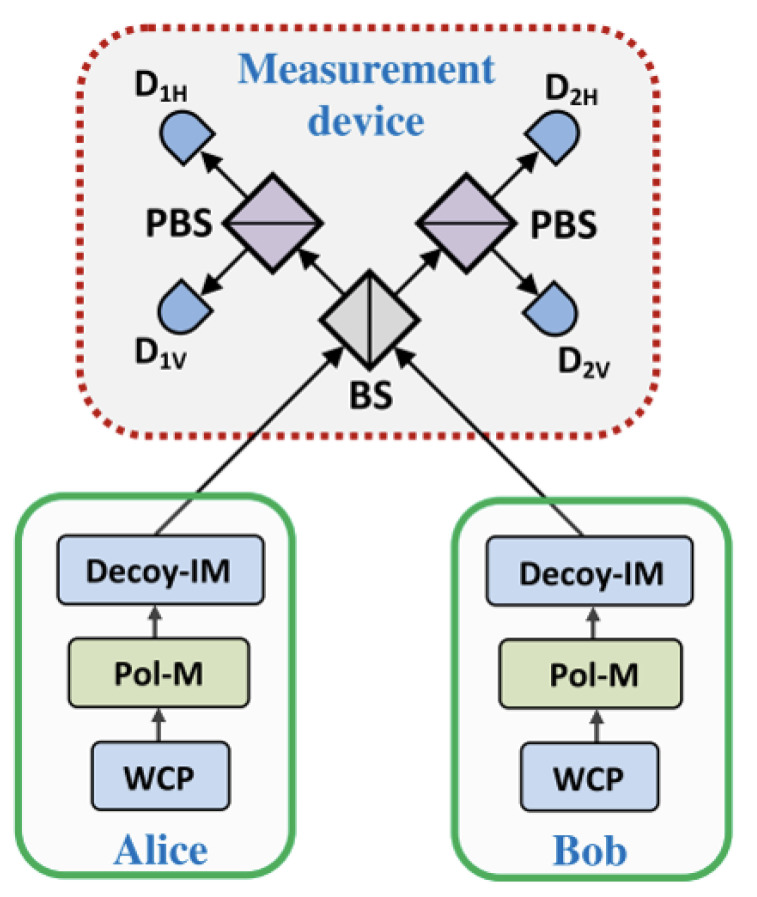
The basic setup of a measurement-device-independent QKD (MDI-QKD) protocol is in Ref. [19]. Alice and Bob use three devices to prepare their photons, and the third party will make a joint measurement and announce measurement output.

Prior to 2012, most quantum cryptographic protocols, including QKD and many two-party cryptography protocols, used single-state measurement. The earliest application of joint measurement to quantum protocols is introduced by Hoi-Kwong Lo [19]. In Ref. [19], he presented the idea of MDI-QKD using joint measurement. The measurement method is shown in Figure 1.

In Figure 1, Alice and Bob will prepare a single quantum state and send it to the third party, Charlie. Charlie will measure those quantum states in Bell basis. The state $|\phi^{-}\rangle =\frac{1}{\sqrt{2}}(|HV\rangle -\left|VH\rangle \right)$ is joint by a click in $D_{1H}$ and $D_{2V}$ or $D_{1V}$ and $D_{2H}$, and $|\phi^{+}\rangle =\frac{1}{\sqrt{2}}(|HV\rangle +\left|VH\rangle \right)$ is joint by a click in $D_{1H}$ and $D_{1V}$ or $D_{2H}$ and $D_{2V}$. Therefore, Alice and Bob can get the raw key based on measurement outcomes and prepared basis, which is shown in Table 1.

**Figure 2:**
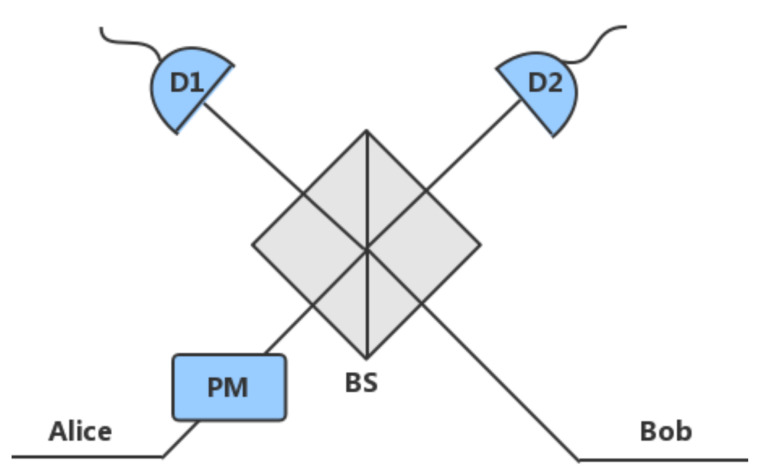
Mesurement setup used in phase-matching QKD (PM-QKD).

Another joint measurement method uses phase coding, which is generally used in the continuous variable QKD. The representative protocols are PM-QKD [22] and TF-QKD [23]. The measurement method is shown in Figure 2. Phase-matching QKD uses coherent state to send information. We define that $\delta_{a}=|\sqrt{\mu_{a}}e^{i(\phi_{a}+\pi k_{a})}\rangle$ and $\delta_{b}=|\sqrt{\mu_{b}}e^{i(\phi_{b}+\pi k_{b})}\rangle$, where $\phi_{a},\phi_{b}\in \left\{0,\frac{\pi }{2}\right\}$ are the basis phase chosen by Alice and Bob.

According to Mach–Zehnder interference, the detector $D_{1}$ clicks when the phase difference of $\delta_{a}$ and $\delta_{b}$ is an even multiple of π, and the detector $D_{2}$ clicks when the phase difference of $\delta_{a}$ and $\delta_{b}$ is an odd multiple of π. When a phase difference of $\delta_{a}$ and $\delta_{b}$ is not a multiple of π, a random click occurs. Bob will flip his key when detector $D_{2}$ click because only $|k_{a}-k_{b}|=1$ will cause the phase difference to be an odd multiple of π.

### 2.3. Error Estimation

Error estimation is one of the most important methods to ensure security in quantum cryptographic protocols. However, so far, in the two-party quantum encryption protocol, no method to improve the security of the protocol by error estimation has been seen. This is due to the asymmetry of the information in the two-party encryption protocols and the coupling between the measurement results and final key. We find that joint measurement reduces this coupling and try to introduce the error estimation method into the two-party encryption protocol. In this paper, because of the asymmetry of the information, we use the random sampling method for error estimation.

In QKD, the operation process of the random sampling method can be described as follows: Among the raw key ${\left(k_{0},\cdots ,k_{l-1}\right)}_{A}$ and ${\left(k_{0},\cdots ,k_{l-1}\right)}_{B}$ owned by Alice and Bob, randomly extract a certain percentage *p* of the key at the corresponding positions and publishing these bits through the classical channel with trusted authentication. The inconsistency rate of the sampling key can be regarded as the code error rate of the raw key (since the extracted key has been published, it cannot be used in subsequent processing steps and needs to be discarded). In the two-party quantum cryptographic protocol, due to the asymmetry of information (for example, in the ROT protocol, after performing base matching, Bob does not discard the key that failed to match, but performs key separation according to his chosen *c*), Alice will perform random sampling from all keys, and require that the preparation base and key of the sampling part be made public, and then calculate the code error rate.

Assume that the error rate of the raw key owned by Alice and Bob is *e* and the key length is *l*, compared with the Alice’s key, Bob’s raw key has el errors. The amount of randomly extracted key bits is pl and satisfies el<pl, that is, e<p. Assume that there are *m* bit errors in the extracted pl keys, then consider that the error rate of the raw key is:

$$e^{\prime }=\frac{m}{pl}.$$

In this paper, in order to ensure the security of the two-party encryption protocol, we put the error estimation process before the base matching. Thus, we can get:

$$e^{\prime }=\frac{\mathrm{num}(x_{i}\neq y_{i}|\theta_{B_{i}}=\theta_{A_{i}})}{\mathrm{num}(\theta_{B_{i}}=\theta_{A_{i}})}.$$

### 2.4. Privacy Amplification

Generally speaking, we will use two-universal hash function for privacy amplification. The definition of two-universal hash function is as follows:

**Definition** **2** (Two-universal hash function). *Let F be a cluster of functions $f:{\left\{0,1\right\}}^{n}\to {\left\{0,1\right\}}^{l}$ (l≤n). If for all $x\neq y\in_{R}{\left\{0,1\right\}}^{n}$, we have:*

$$Pr_{f\in_{R}F}\left[f\left(x\right)=f\left(y\right)\right]\leq 2^{-l}.$$

*Then we say that F is two-universal.*

Using two-universal hash function for privacy amplification, we also have privacy amplification theorem [2].

Firstly, we know the security of a key is defined with respect to its L1-distance from a perfect key which is uniformly distributed and independent of the adversary’s state. Then the L1-distance from uniform of $\rho_{XQ}$ given Q is:

$$d\left(\rho_{XQ}|Q\right):=\left|\right|\rho_{XQ}-\rho_{U}⊗\rho_{Q}\left|\right|$$

where $\rho_{U}$ is the fully mixed state.

**Theorem** **2** (Privacy amplification [25]). *Given a set of two-universal hash functions $F:{\left\{0,1\right\}}^{n}⊗R\to {\left\{0,1\right\}}^{l}$, and a hash function $F\in_{R}F$, let $\rho_{XQ}$ be a classical-quantum state, then for any ε≥0. we have:*

$$d\left[F\left(X\right)|F,Q\right]\leq 2^{-\frac{1}{2}\left[H_{\min}^{\varepsilon }\left(X|Q\right)-l\right]-1}+\varepsilon .$$

## 3. Weak String Erasure

In order to better demonstrate the application of joint measurement and error estimation technology in two-party cryptographic protocols, we first discuss its enhancement to the security performance of weak string erasure (WSE), which was originally proposed by Konig [2], and studied as the basic protocol of other two-party cryptographic protocols.

### 3.1. Definition

**Figure 3:**
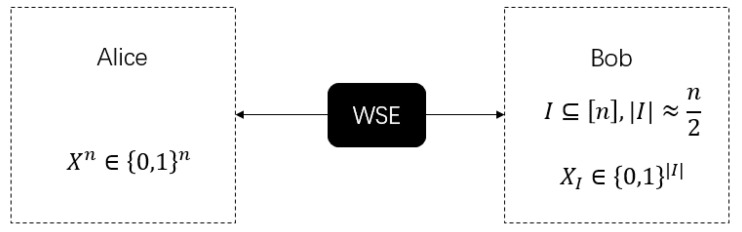
The ideal functionality of weak string erasure (WSE).

Before introducing our redesigned WSE protocol, we first introduce its definition. WSE is a basic two-party cryptographic protocol between Alice and Bob that can be used to construct other two-party cryptographic protocols, such as bit commitment, oblivious transfer, etc. The ideal functionality of WSE is shown in Figure 3 [2].

The process of WSE can be seen as a black box, with no inputs from Alice and Bob. As outputs, Alice gets a randomly chosen bits string $X^{n}$ and Bob obtains a randomly chosen subset of indices I⊆[n] and the bits $X_{I}\in {\left\{0,1\right\}}^{\left|I\right|}$. Next, we denote *A* and *B* as honest Alice and Bob, and $A^{\prime }$ and $B^{\prime }$ as dishonest Alice and Bob. ρ represents the joint state generated in the actual protocol operation, and σ represents the state generated in the ideal protocol operation.

The specific definition of WSE is as follows [2]:

**Definition** **3** (Weak string erasure [2]). *A (n,λ,ε)-weak string erasure (WSE) scheme is a protocol between Alice and Bob satisfying the following properties:*

*1*. Correctness: If both parties are honest, then for any attack strategy of the third-party attacker, Alice always gets a uniformly distributed string $X^{n}\in_{R}{\left\{0,1\right\}}^{n}$ and Bob will get an index I∈[n] and $X_{I}\in {\left\{0,1\right\}}^{\left|I\right|}$;
*2*. *Security for Alice: If Alice is honest, then for any attack strategy of dishonest Bob, we have:*
  $$\frac{1}{n}H_{\min}^{\varepsilon }\left(X^{n}|B^{\prime }\right)\geq \lambda .$$
*3*. *Security for Bob: If Bob is honest, then for any attack strategy of dishonest Alice, there exists α≥0:*
  $$H_{\min}\left(I|A^{\prime }\right)\geq \alpha .$$

### 3.2. Protocol

In the previous protocol, there is a no error estimation process because the measurement results of the BB84 protocol are directly related to the final key. We redesign the WSE protocol by using the independence of key and measurement results of the MDI-QKD and PM-QKD protocols, adding a error estimation process to improve the security of the protocol.

The specific agreement is as follows:

1. Alice chooses a string $x^{n}\in_{R}{\left\{0,1\right\}}^{n}$ and bases the specifying string $\theta_{A}^{n}\in_{R}{\left\{+,\times \right\}}^{n}$ randomly. She encodes each bit $x_{i}$ in the basis given by $\theta_{A_{i}}$ (as $H^{\theta_{A_{i}}}|x_{i}\rangle$) and sends it to the third party Charlie;
2. Similarly to Alice, Bob chooses a string $y^{n}\in_{R}{\left\{0,1\right\}}^{n}$ and bases specifying string $\theta_{B}^{n}\in_{R}{\left\{+,\times \right\}}^{n}$ randomly. He encodes each bit $y_{i}$ in the basis given by $\theta_{B_{i}}$ (as $H^{\theta_{B_{i}}}|y_{i}\rangle$), and sends it to the third party Charlie;
3. Charlie performs a Bell measurement, and announces the outcome;
4. Alice selects a subset of the measurement outcome as the error estimator (about *m* qubits) and sends a subset of the measurement outcome $I_{check}$ to Bob. Bob sends $\theta_{B_{check}}$ and a subset of the measurement outcome $y_{check}$ ($y_{check},\theta_{B_{check}}=\left\{y_{i},\theta_{B_{i}}|i\in I_{check}\right\}$) to Alice. Then, they initiate error estimation process and compute:
  $$Q_{u}=\frac{\mathrm{num}(x_{i}\neq y_{i}|\theta_{B_{i}}=\theta_{A_{i}})}{\mathrm{num}(\theta_{B_{i}}=\theta_{A_{i}})};$$
5. If $Q_{u}>e_{r}$, the communication is terminated, otherwise, the process continues;
6. Alice sends the remaining bases $\theta_{A}^{n-m}$ to Bob and outputs the remaining string $x^{n-m}$;
7. Bob computes $I:=\{i\in \left[n\right]|i\notin I_{check}\wedge \theta_{A_{i}}=\theta_{B_{i}}\}$ and outputs $\left(I,z^{\left|I\right|}\right):=\left(I,y_{I}\right)$.

### 3.3. Security Proof of WSE

Before analyzing the security of WSE protocol, we need to explain the constraint of Bob’s storage capacity under joint state measurement and error estimation. When we remove any assumption about storage devices, we need other approaches to limit Bob’s ability to store quantum states sent by Alice. Due to the constraints of the protocol process, we naturally think that Bob would cause the error rate increasement of the final key when he stores the quantum state and the error estimation is used to detect this attack. Next, we need to explain an important conception of the error correction upper bound of any channel error correction code. From [26] we know that:

$$f=\frac{1-R}{h(e)}$$

where *f* is the reconciliation efficiency which is given by the redundancy of disclosed information to the theoretical limit necessary for successful error correction, *R* is the code rate of a given channel error correction code, *e* is the error rate, and function *h* is the Shannon binary entropy. Then we can get the error correction upper bound when *f* approaches 1, i.e., its Shannon limit:

$$e_{r}=\lim_{f\to 1}e=\lim_{f\to 1}h^{-1}\left(\frac{1-R}{f}\right).$$

where $h^{-1}$ is the inverse function of *h*.

We consider when Bob stores the quantum state because the joint measurement cannot be performed and the published detection results are random. The increasement of error rate is explained the Lemma 3.

**Lemma** **3.** *Assume that Bob has a perfect and unlimited capacity of quantum memory. Our protocol has a storage rate v, where $v\leq 2e_{r}$.*

**Proof.**  In our protocol, the measurement outcomes are jointly measured by a third party in the bell state and published before Alice sends the bases $\theta_{A}$. Alice will ask Bob to publish partial information for error estimation before sending bases $\theta_{A}$. Now, we assume that Bob’s storage rate is *v*, which means Bob will store vn quantum state in his memory. If Bob stores the quantum states, it means that he can not measure these quantum states, because quantum mechanics tells us that the measurement will cause the collapse of the quantum states and the loss of information. Therefore, Bob can publish a random fake outcome, and we have error rate introduced by this:

$$Q_{u}=\frac{n\left(1-v\right)e_{c}+\frac{1}{2}nv}{n}\leq e_{r},$$

and we have:

$$v\leq 2e_{r},$$

where $e_{c}$ is error rate that caused by channel noise. ☐

In fact, with Lemma 3, we can easily convert our protocol into a WSE protocol under the bounded-storage model. Therefore, we can use the proof methods and results in Ref. [2,17] to prove the security of our protocol.

**Lemma** **4** (Security for Alice). *Fix $\delta \in [0,\frac{1}{4}]$, and let,*

$$\varepsilon =2\exp\left(-\frac{{\left(\frac{\delta }{4}\right)}^{2}}{32{\left(2+\log_{2}\frac{4}{\delta }\right)}^{2}}\right),$$

*then for any attack strategy of dishonest Bob with any storage model $F:B\left(H_{\mathrm{in}}\right)\to B\left(H_{\mathrm{out}}\right)$, we have:*

$H_{\min}^{\varepsilon }{\left(X^{n}|B^{\prime }\right)}_{\sigma }\geq n\left(\frac{1}{2}-\delta -v\right)>0.$

**Proof.**  According to the conclusion in Ref. [2], we have:

$$\frac{1}{n}H_{\min}^{\varepsilon }{\left(X^{n}|B^{\prime }\right)}_{\sigma }\geq -\frac{1}{n}logP_{\mathrm{succ}}^{F}\left(\left(\frac{1}{2}-\delta \right)n\right)\geq v\gamma^{N}\left(\frac{\frac{1}{2}-\delta }{v}\right),$$

where we have:

$$\begin{array}{cc} \gamma^{N}\left(R\right) & =\max_{\alpha \geq 1}\frac{\alpha -1}{\alpha }\left\{R-\log_{2}d+\frac{1}{1-\alpha }\log_{2}\left[{\left(r+\frac{1-r}{d}\right)}^{\alpha }+\left(d-1\right){\left(\frac{1-r}{r}\right)}^{\alpha }\right]\right\}, \end{array}$$

and in our protocol, we have parameters $\delta \in [0,\frac{1}{4}]$, $v=2e_{r}$,$C_{N}=1$, r=1, and d=2. So, we have:

$$\gamma^{N}\left(R\right)=\max_{\alpha \geq 1}\frac{\alpha -1}{\alpha }\left(R-1\right),$$

then,

$$\begin{array}{cc} H_{\min}^{\varepsilon }{\left(X^{n}|B^{\prime }\right)}_{\delta } & \geq nv\gamma^{N}\left(\frac{\frac{1}{2}-\delta }{v}\right)\\ & =nv\max_{\alpha \geq 1}\frac{\alpha -1}{\alpha }\left(\frac{\frac{1}{2}-\delta }{v}-1\right)\\ & \geq n\left(\frac{1}{2}-\delta -v\right)\geq 0. \end{array}$$

☐

Next, we will discuss the security for Bob. Proving the security for Bob is relatively simple because Bob has no other leaked information besides his quantum state information during the protocol.

**Lemma** **5** (Security for Bob). *According to [2,27], for any attack of dishonest Alice with any storage model $F:B\left(H_{\mathrm{in}}\right)\to B\left(H_{\mathrm{out}}\right)$, then we have:*

$$H_{\min}\left(y^{n}|A^{\prime }\right)\geq -n\log_{2}\left(\frac{1}{2}+\frac{1}{2\sqrt{2}}\right),$$

## 4. 1-2 Random Oblivious Transfer

In this section, we further investigate 1-2 random oblivious transfer (ROT), which is also a basic two-party cryptographic protocol as WSE. Similarly, we give its definition first and then propose our protocol based on joint measurement and error estimation followed by its security proof.

### 4.1. Definition

**Figure 4:**
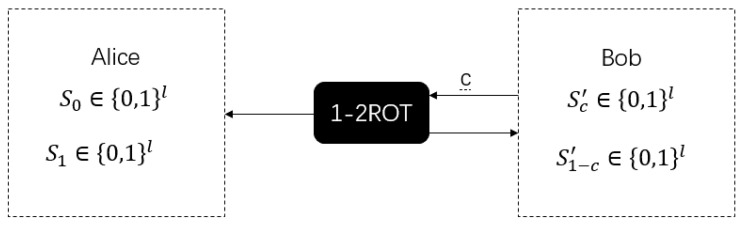
The ideal functionality of 1-2 random oblivious transfer (ROT). Bob has input *c*, Alice gets $S_{0},S_{1}$, and Bob gets outputs $S_{c}^{\prime },S_{1-c}^{\prime }$ with $S_{c}=S_{c}^{\prime }$ and $S_{1-c}\neq S_{1-c}^{\prime }$.

As in Figure 4, like the WSE protocol, the 1-2 random oblivious transfer (ROT) protocol is also a basic two-party cryptographic protocol and is a random version of the 1-2 oblivious transfer (OT). Based on the 1-2 ROT protocol, we can easily implement the 1-2 OT protocol and the bit commitment (BC) protocol. In the 1-2 ROT protocol, instead of inputting two information strings $m_{0},m_{1}\in {\left\{0,1\right\}}^{l}$, Alice obtains two random key strings $S_{0},S_{1}\in {\left\{0,1\right\}}^{l}$. At the same time, Bob obtains the random key string $S_{c}$ according to its input *c*. If we want to implement the 1-2 OT protocol, just after running the 1-2 ROT protocol, Alice encrypts the information strings $m_{0}$ and $m_{1}$ with the two strings of keys $S_{0}$ and $S_{1}$ obtained by ROT protocol. Bob can use $S_{c}$ for decryption to obtain $m_{c}$.

In the security definition of the 1-2 ROT protocol, Alice cannot obtain Bob’s input *c*, and Bob cannot obtain another string of keys $S_{1-c}$ except $S_{c}$. The specific definition of security is as follows:

**Definition** **4.** *An ε - secure 1-2 ROT is a protocol between Alice and Bob, where Bob has input c∈{0,1}, and Alice has no input, satisfying:*

*1*. *Correctness: If Alice and Bob are honest, then for any distribution of Bob’s input c which is unknown to Alice, Alice gets outputs $S_{0},S_{1}\in {\left\{0,1\right\}}^{l}$ which are ε-close to randomness and independent of c, and Bob obtains $Y=S_{c}$ with probability ε;*
*2*. *Security for Alice: If Alice is honest, then for any cheating strategy of Bob resulting in his state $\rho_{B}$, there exists a random variable D∈{0,1}, and λ>0 such that:*
  $$H_{\min}\left(S_{1-D}|B^{\prime }\right)\geq \lambda ,$$
  *and*
  $$d(S_{1-D}|B^{\prime })\leq \varepsilon ;$$
*3*. *Security for Bob: If Bob is honest and obtains output Y, then for any cheating strategy of Alice resulting in her state $\rho_{A}$, there exists a random variable D∈{0,1}, such that:*
  $$H_{\min}\left(D|A^{\prime }\right)\geq 1-\varepsilon ,$$
  *and*
  $$\mathrm{Pr}(Y=S_{c}^{\prime })\leq \varepsilon .$$

### 4.2. Protocol

We now give the specific 1-2 ROT protocol using error estimation as follows:

1. Preparation: Alice chooses $x^{n}\in_{R}{\left\{0,1\right\}}^{n}$ and $\theta_{A}^{n}\in_{R}{\left\{+,\times \right\}}^{n}$, and Bob chooses $y^{n}\in_{R}{\left\{0,1\right\}}^{n}$ and $\theta_{B}^{n}\in_{R}{\left\{+,\times \right\}}^{n}$. Both parties send the encoding quantum state ${|x\rangle }_{\theta_{A}}^{n}$ or ${|y\rangle }_{\theta_{B}}^{n}$ to third party Charlie;
2. Measurement: Charlie measures ${|x\rangle }_{\theta_{A}}^{n}$ and ${|y\rangle }_{\theta_{B}}^{n}$ with Bell measurement, and announces the outcome;
3. Error estimation: Alice chooses $I_{check}\in_{R}2^{\left[n\right]}$ and $|I_{check}|=m$, and sends $I_{check}$ to Bob. Bob sends $y_{check}$, and $\theta_{B_{Check}}=\left\{y_{i},\theta_{B_{i}}|i\in I_{check}\right\}$ to Alice. Then Alice calculates the error rate:
  $$Q_{u}=\frac{\mathrm{num}(x_{i}\neq y_{i}|\theta_{B_{i}}=\theta_{A_{i}})}{\mathrm{num}(\theta_{B_{i}}=\theta_{A_{i}})}.$$
  If $Q_{u}>e_{r}$, they stop communication, otherwise they continue where $e_{r}$ is the error correction upper bound;
4. Key division: Both parties discard the data that used in error estimation. Alice sends $\theta_{A}^{n-m}$ to Bob, Bob divides the key according to $\theta_{A}^{n-m}$, $\theta_{B}^{n-m},$ where $I_{c}=\left\{i|\theta_{A_{i}}=\theta_{B_{i}}\right\}$ and $I_{1-c}=\left\{i|\theta_{A_{i}}\neq \theta_{B_{i}}\right\}.$ Bob sends $I_{0},I_{1}$ to Alice;
5. Post processing: Alice chooses two hash function $f_{0},f_{1}\in_{R}F_{h}$, and calculates $syn\left(X|I_{0}\right),syn\left(X|I_{1}\right)$. Alice passes $f_{0}$, $f_{1}$, $syn(X|I_{0})$, and $syn(X|I_{1})$ to Bob. Bob corrects the errors and outputs $S_{c}=f_{c}\left(Y|I_{c}\right)$. Alice outputs $S_{0}=f_{0}\left(X|I_{0}\right)$ and $S_{1}=f_{1}\left(X|I_{1}\right)$.

### 4.3. Security Proof of 1-2 ROT

According to the definition, we will prove the security of our proposed ROT protocol from the perspective of correctness, security for Alice, and security for Bob successively.

For correctness, if both parties are honest, Bob can calculate $I_{0},I_{1}$ according to *c*, and $S_{c}$, and Alice can also get $S_{0},S_{1}$. The focus is mainly on security for Alice and Bob.

**Lemma** **6** (Security for Alice). *In 1-2 ROT protocol, n represents the number of bits transmitted during the protocol. $\sigma_{B^{\prime }X^{n}}$ represents the state generated in the ideal protocol operation which consists of dishonest Bob and the variable $X^{n}$ of n transmitted bits. $\rho_{X_{n}B^{\prime }}$ represents the joint state generated in the actual protocol operation which consists of dishonest Bob and the variable $x_{n}$ of n transmitted bits. If Alice is honest, n→∞ and the trace distance between these two states $\left|\right|\sigma_{B^{\prime }X^{n}}-\rho_{B^{\prime }X^{n}}\left|\right|\leq \varepsilon$ with $\varepsilon =2\exp\left(-\frac{\delta^{2}}{32{\left(2+\log_{2}\delta \right)}^{2}}\right)$. Then we fix $\delta \in \{0,\frac{1}{4}\}$, we can get:*

*1) $H_{\min}\left(S_{1-D}|B^{\prime }\right)\geq \left(\frac{1}{4}-\delta -v\right)n-1$,*

*2) $l\leq \left(\frac{1}{4}-\delta -2e_{r}\right)n+1-\log_{2}\frac{1}{\epsilon^{2}}$.*

**Proof.**  With uncertainty relation theorem, we have:

$$H_{\min}^{\varepsilon }\left(X^{n}|M\theta_{A}^{n}\right)\geq \left(\frac{1}{2}-2\delta \right)n,$$

where *M* is the outcome that announced by Charlie. According to entropy sampling theorem:

$$H_{\min}^{\varepsilon }\left(X_{1-D}|DM\theta_{A}^{n}\right)\geq \left(\frac{1}{4}-\delta \right)n-1,$$

and in our protocol, according to Lemma 3, we have the storage rate $v=2e_{r}$, then:

$$H_{\min}^{\varepsilon }\left(X_{1-D}|DM\theta_{A}^{n}Q\left(\rho_{A}\right)\right)\geq H_{\min}^{\varepsilon }\left(X_{1-D}|DM\theta_{A}^{n}\right)-vn=\left(\frac{1}{4}-\delta -v\right)n-1$$

By using privacy amplification theorem:

$$d\left(f_{1-D}\left(S_{1-D}\right)|D\theta_{A}f_{D}\rho_{A}MQ\left(\rho_{A}\right)\right)\leq 2^{-\frac{1}{2}\left[\left(\frac{1}{4}-\delta -2e_{r}\right)-1-l\right]-1}+\varepsilon ,$$

and let the above formula be less than 2ε, we can get:

$$l\leq \left(\frac{1}{4}-\delta -2e_{r}\right)n+1-\log_{2}\frac{1}{\varepsilon^{2}}.$$

☐

**Lemma** **7** (Security for Bob). *In 1-2 ROT protocol, n represents the number of bits transmitted during the protocol. $\sigma_{A^{\prime }c}$ represents the state generated in the ideal protocol operation which consists of dishonest Alice and commit bit c. $\rho_{A^{\prime }}⊗\tau \left\{0,1\right\}$ represents the joint state generated in the actual protocol operation which consists of dishonest Alice and commit bit c that is uniformly distributed on {0,1}. If Bob is honest, n→∞ and the trace distance between these two states $\left|\right|\left(\sigma_{A^{\prime }c}\right)-\rho_{A^{\prime }}⊗\tau \left\{0,1\right\}\left|\right|\leq \varepsilon$, and there exits ε≥0, then the conditional entropy with respect to c and $A^{\prime }$, we have:*

*(1) $H(c|A^{\prime })\geq 1-\varepsilon$*

**Proof.**  According to the definition of ROT protocol, if Alice is dishonest, then her purpose is to get *c* chosen by Bob. In our protocol, Bob’s information leakage to Alice are $\rho_{B},y_{check},\theta_{check},I_{0}$ and $I_{1}$. We have:

$$\mathrm{Pr}\left(c|y_{check}\theta_{check}I_{0}I_{1}\rho_{B}\right)=\mathrm{Pr}\left(c|I_{0}I_{1}\rho_{B}\right).$$

As $\mathrm{Pr}(c|I_{0}I_{1}x^{n}y^{n})=1$, we can argue that:

$$\mathrm{Pr}\left(c|I_{0}I_{1}\rho_{B}\right)=\mathrm{Pr}\left(y^{n}|I_{0}I_{1}\rho_{B}\right)=\max\left(\mathrm{Pr}\left(y^{n}|\rho_{B}\right),\frac{1}{2}\right),$$

and with the uncertainty relation theorem:

$$H\left(y^{n}|\rho_{B}\right)=-n\log_{2}\left(\frac{1}{2}+\frac{1}{2\sqrt{2}}\right),$$

we can get:

$$\mathrm{Pr}\left(y^{n}|\rho_{B}\right)=2^{-H(y^{n}|\rho_{B})}={\left(\frac{1}{2}+\frac{1}{2\sqrt{2}}\right)}^{n}.$$

when n→∞, $\mathrm{Pr}\left(c|I_{0}I_{1}\rho_{B}\right)=\max\left(\mathrm{Pr}\left(y^{n}|\rho_{B}\right),\frac{1}{2}\right)=\frac{1}{2}$, so we can get $H\left(c|A^{\prime }\right)=-\sum_{k=0,1}p\left(c=k|A^{\prime }\right)\log_{2}p\left(c=k|A^{\prime }\right)=1$. Namely, exists ε≥0, $H(c|A^{\prime })\geq 1-\varepsilon$. ☐

## 5. Applications for Two Party Cryptography

In this section, we redesign two specific two-party cryptographic protocols using a joint measurement method and briefly analyze their security. The first protocol is bit commitment which is proposed by [1]. The second protocol is password-based identification, which allows us to use passwords for authentication without revealing passwords.

### 5.1. Bit Commitment

In this subsection, we redesign bit commitment protocol using joint measurement and prove the security of this protocol. Quantum bit commitment protocol is one of the earliest proposed quantum two-party encryption protocols. The original version of quantum bit commitment is a variant of quantum coin tossing proposed by Bennett and Brassard [1]. In fact, quantum bit commitment is easy to adapt from 1-2 ROT protocol.

#### 5.1.1. Definition and Protocol

Informally, a standard bit commitment scheme consists of two sub-protocols called commitment protocol and revealing protocol. First, Alice and Bob execute the commitment protocol. Alice has commit bit c∈{0,1} as input, and Bob has no input. As a result of this protocol, Bob will get some evidence about *c*. In the second phase, Alice and Bob execute the revealing protocol, where Alice has an input for remaining evidence and commit bit *c* and Bob also has no input. At the end of this protocol, Bob will output accept or reject according to Alice’s inputs from the commitment protocol and revealing protocol.

If both parties are honest, Bob always accepts the bit *c*. If Alice is dishonest, however, Bob should not output accept. If Bob is dishonest, he should not be able to gain any information about *c* before the revealing protocol is executed. The definition of security in bit commitment protocol is as follows.

**Definition** **5** (Bit commitment [17]). *An ε-secure bit commitment is a protocol between Alice and Bob, where Alice has input c∈{0,1}, and Bob has no input.*

*1*. Correctness: If both parties are honest, then the ideal state $\delta_{cans}$ is defined as:
  *The distribution of commit bit c for Bob is uniform when Bob gets no information about distribution of c besides the information leakage by this protocol, and Bob accepts the commitment:*
  $$\delta_{cans}=\tau_{\left\{0,1\right\}}⊗|\mathrm{accept}\rangle \langle \mathrm{accept}|.$$
*2*. *Security for Alice (ε-hiding): If Alice is honest, then for any joint state $\rho_{cB^{\prime }}$ created by the commit protocol, Bob does not learn c. Here,*
  $$\rho_{cB^{\prime }}\approx_{\varepsilon }\tau_{\left\{0,1\right\}}⊗\rho_{B^{\prime }},$$
  *and the entropy of c:*
  $$H_{\min}\left(c|B^{\prime }\right)\geq 1-\varepsilon .$$
*3*. *Security for Bob (ε-Binding): If Bob is honest, then there exists an ideal cq-state $\delta_{cA^{\prime }V}$ such that for all operations for $\rho_{A}^{\prime }$, we have:*
  $$\mathrm{Pr}[\mathrm{outputs}=\mathrm{accept}|A^{\prime }]\leq \varepsilon .$$

We have rewritten the QBC agreement based on the contents of the ROT agreement as shown below.

Bit commitment - commit phase: The input is commit bit c∈{0,1} for Alice. The output are $S_{c}\in {\left\{0,1\right\}}^{l}$ to Alice, and $S_{0}^{\prime },S_{1}^{\prime }\in {\left\{0,1\right\}}^{l}$ to Bob.

1. Preparation: Alice chooses $x^{n}\in_{R}{\left\{0,1\right\}}^{n}$ and $\theta_{A}^{n}\in_{R}{\left\{+,\times \right\}}^{n}$ furthermore, Bob chooses $y^{n}\in_{R}{\left\{0,1\right\}}^{n}$ and $\theta_{B}^{n}\in_{R}{\left\{+,\times \right\}}^{n}$. Both parties send the encoding quantum state ${|x\rangle }_{\theta_{A}}^{n}$ or ${|y\rangle }_{\theta_{B}}^{n}$ to the third party Charlie;
2. Measurement: Charlie measures ${|x\rangle }_{\theta_{A}}^{n}$ and ${|y\rangle }_{\theta_{B}}^{n}$ with Bell basis, and announces the outcome;
3. Error estimation: Bob chooses $I_{check}\in_{R}2^{\left[n\right]}$ and $|I_{check}|=m$, and sends $I_{check}$ to Alice. Alice sends $x_{check},\theta_{A_{Check}}=\left\{x_{i},\theta_{A_{i}}|i\in I_{check}\right\}$ to Bob. Bob calculates the error rate $Q_{u}$:
  $$Q_{u}=\frac{\mathrm{num}(x_{i}\neq y_{i}|\theta_{B_{i}}=\theta_{A_{i}})}{\mathrm{num}(\theta_{B_{i}}=\theta_{A_{i}})}.$$
  If $Q_{u}>e_{r}$, they stop communication, else they continue. Here $e_{r}$ is error correction upper bound;
4. Key division: Both parties discard the bits that used in error estimation. Bob sends $\theta_{B}^{n-m}$ to Alice. Alice divides the key according to $\theta_{A}^{n-m}$, $\theta_{B}^{n-m}$, where $I_{c}=\left\{i|\theta_{A_{i}}=\theta_{B_{i}}\right\}$ and $I_{1-c}=\left\{i|\theta_{A_{i}}\neq \theta_{B_{i}}\right\}$, and sends $I_{0},I_{1}$ to Bob;
5. Post processing: Bob chooses two hash functions $f_{0},f_{1}\in_{R}F_{h}$, and calculates two syndromes $\mathrm{syn}\left(X|I_{0}\right),\mathrm{syn}\left(X|I_{1}\right)$. Bob sends $f_{0},f_{1},\mathrm{syn}\left(X|I_{0}\right),\mathrm{syn}\left(X|I_{1}\right)$ to Alice. Alice corrects errors and outputs $S_{c}=f_{c}\left(Y|I_{c}\right)$. Bob outputs $S_{0}^{\prime }=f_{0}\left(X|I_{0}\right),S_{1}^{\prime }=f_{1}\left(X|I_{1}\right)$.

Bit commitment–revealing phase: The input is $S_{c}$ for Alice. The outputs are c∈{0,1} and ans∈{accept,reject} to Bob.

1. Alice: Alice sends $S_{c}$ and *c* to Bob;
2. Bob: If $S_{c}=S_{c}^{\prime }$, then Bob obtains *c* and ans=accept. Otherwise, he outputs ans=reject.

#### 5.1.2. Security Analysis

The correctness of the protocol does not need to be proven because the protocol is designed according to the definition of bit commitment protocol. Its ε-hiding is guaranteed by the security of the ROT protocol.

**Lemma** **8** (Security for Alice). *n represents the number of bits transmitted during the protocol. Let n→∞, we have:*

*(1) $\delta_{cB^{\prime }}\approx_{\varepsilon }\tau_{\left\{0,1\right\}}⊗\rho_{B^{\prime }},$*

*(2) $H_{\min}\left(c|B^{\prime }\right)\geq 1-\varepsilon .$*

**Proof.**  Our Commitment protocol is adopted from the 1-2 ROT, and according to Definition 5, we have $H_{\min}\left(c|B^{\prime }\right)\geq 1-\varepsilon .$ ☐

**Lemma** **9** (Security for Bob). *n represents the number of bits transmitted during the protocol. Fix $\delta \in [0,\frac{1}{4}]$, and exist ε→0, we have:*

*$\mathrm{Pr}(\mathrm{ans}=\mathrm{accept}|A^{\prime })\leq \varepsilon$.*

**Proof.**  According to Lemma 5,

$$H_{\min}\left(y^{n}|A^{\prime }\right)\geq -n\log_{2}\left(\frac{1}{2}+\frac{1}{2\sqrt{2}}\right),$$

because

$$\mathrm{Pr}\left(y^{n}|A^{\prime }\right)=2^{-H_{\min}\left(y^{n}|A^{\prime }\right)}\leq {\left(\frac{1}{2}+\frac{1}{2\sqrt{2}}\right)}^{n}.$$

we can easily get $\mathrm{Pr}\left(\mathrm{ans}=\mathrm{accept}\right)|A^{\prime })\leq \varepsilon$. ☐

### 5.2. Password-Based Identification

In this subsection, we introduce the joint measurement method to password-based protocol from [15].

Password-based identification (PID) so far is one of the most widely-used authentication methods. In this protocol, the user and server share a series of keys and the user logs in the system server by verifying the keys. Its security definition contains two points. The first is that users who do not know the password cannot log into the system server successfully and cannot learn other users’ password through this protocol. The second is that the dishonest server (eg. scam server) cannot learn the password holds by honest users. For the convenience of description, in the following, we use Alice instead of user and Bob instead of server. Formally, security is defined as follows.

**Definition** **6** ((n,λ,ε)-secure PID). *An (n,λ,ε)-secure PID is a protocol between Alice and Bob, where Alice and Bob has input password $w\in {\left\{0,1\right\}}^{l}$.*

*1*. Correctness: If both parties are honest, Bob will always output "accept" at the end of the protocol;
*2*. *Security for Alice: If Alice is honest, then for any cheating strategy of Bob resulting in his state $\rho_{B}$, we have λ≥0, and:*
  $$H_{\min}^{\varepsilon }\left(w|B^{\prime }\right)\geq \lambda ;$$
*3*. *Security for Bob: If Bob is honest, then for any cheating strategy of Alice resulting in her state $\rho_{A}$, there exists ε≥0, we have:*
  $$\mathrm{Pr}(\mathrm{outputs}=\mathrm{accept}|A^{\prime })\leq \varepsilon .$$

Next, we give our PID protocol. The input is $w\in {\left\{0,1\right\}}^{l}$ for Alice and the output is ans∈{accept,reject} for Bob.

1. Preparation: Alice chooses $x^{n}\in_{R}{\left\{0,1\right\}}^{n}$ and $\theta_{A}^{n}\in_{R}{\left\{+,\times \right\}}^{n}$, and Bob also chooses $y^{n}\in_{R}{\left\{0,1\right\}}^{n}$ and $\theta_{B}^{n}\in_{R}{\left\{+,\times \right\}}^{n}$. Both parties send the encoding quantum state ${|x\rangle }_{\theta_{A}}^{n}$ or ${|y\rangle }_{\theta_{B}}^{n}$ to the third party, Charlie;
2. Measurement: Charlie measures ${|x\rangle }_{\theta_{A}}^{n}$ and ${|y\rangle }_{\theta_{B}}^{n}$ with Bell measurement, and announces the outcome;
3. Error estimation: Alice chooses $I_{check}\in_{R}2^{\left[n\right]}$ and $|I_{check}=m$, and sends $I_{check}$ to Bob. Bob sends $y_{check},\theta_{B_{Check}}=\left\{y_{i},\theta_{B_{i}}|i\in I_{check}\right\}$ to Alice. Alice calculates the error rate $Q_{u}$:
  $$Q_{u}=\frac{\mathrm{num}(x_{i}\neq y_{i}|\theta_{B_{i}}=\theta_{A_{i}})}{\mathrm{num}(\theta_{B_{i}}=\theta_{A_{i}})}.$$
  If $Q_{u}>e_{r}$, they stop communication, else they continue. Here, $e_{r}$ is the error correction upper bound;
4. Key shifting: Bob calculates a string $\kappa \in {\left\{0,1\right\}}^{n}$ such that $\kappa =c\left(w\right)⊕\theta_{B}^{n}$( $\kappa_{i}=0$ means basis is +, anyone else). He sends the string κ to Alice, and they define the shifted code ${\hat{\theta }}_{B}^{n}=c\left(w\right)⊕\kappa$. Alice sends $\theta_{A}^{n}$ and a hash function $f\in_{R}F$ to Bob. Both computes $I_{w}=\left\{i|\theta_{A_{i}}={\hat{\theta }}_{B_{i}}\right\}$;
5. Identification: Bob sends $g\in_{R}G$ to Alice. Alice sends $z=f\left(x|I_{w}\right)⊕g\left(w\right)$ to Bob. Bob accepts if and only if $z=f\left(y|I_{w}\right)⊕g\left(w\right)$.
  We omit the proof part because the process is roughly similar to Ref. [17].

## 6. Conclusions

In this paper, we proposed several two-party cryptographic protocols based on joint measurement and error estimation, including WSE, 1-2 ROT, and other protocols, and demonstrated their security. Compared with the protocol mentioned in [2,17,28,29], our protocols discarded the assumption that the attacker’s storage device was defective, but instead employed a combination of joint measurement and error estimation to limit the quantum storage of the attacker. Our protocols had no assumptions, were more secure, and had wider applicability. The two basic two-party cryptographic protocols mentioned in this paper could easily be extended to other two-party encryption protocols, such as 1-2 OT and quantum identification protocols.

We eliminated the assumption that the attack was bounded by the attacker’s technology, and employed the technique of joint measurement and error estimation to improve two basic quantum two-party cryptographic protocols. We demonstrated that our improved protocols offered stronger security and is applicable to many specific quantum two-party cryptographic protocols such as BC and PID.

Inspired by [30,31], we learned that quantum coherence plays an important role in quantum key distribution and quantum random number generation, and this might also be used to improve our work. Future work will also begin with this aspect. 

## Figures and Tables

**Table 1:** Alice or Bob flip their key based on the outcomes of measurement and announced prepared basis [19].

Alice & Bob Basis	Relay Output $|\phi^{-}\rangle$	Relay Output $|\phi^{+}\rangle$
+	Bit flip	Bit flip
×	Bit flip	No bit flip
